# Left atrial reentrant tachycardia with interatrial dissociation mimicking accelerated idioventricular rhythm in a patient with a cardiac resynchronization defibrillator

**DOI:** 10.1016/j.hrcr.2021.06.007

**Published:** 2021-06-29

**Authors:** Kyoichiro Yazaki, Daigo Yagishita, Morio Shoda, Shohei Kataoka, Koichiro Ejima, Nobuhisa Hagiwara

**Affiliations:** Department of Cardiology, Tokyo Women’s Medical University, Tokyo, Japan

**Keywords:** Atrial tachycardia, Cardiac resynchronization therapy, Interatrial conduction disturbance, Ventriculoatrial dissociation, Wide QRS arrhythmia

## Introduction

In heart failure (HF) patients with reduced left ventricular systolic function, the management of arrhythmias is crucial. It has been estimated that 33% of these patients suffer from ventricular arrhythmias, which are associated with an increased mortality.^1^ Furthermore, supraventricular arrhythmias could also be associated with significant worsening of the HF status.^2^ Cardiac resynchronization therapy (CRT), an advanced cardiac implantable device therapy to restore the electrical/mechanical dyssynchrony, may be interrupted during those tachyarrhythmias; therefore, a reduction in the biventricular pacing rate may lead to a poor prognosis.^3^^,^^4^ Thus, a correct diagnosis and subsequent treatment of those arrhythmias are essential in HF patients with CRT. Herein, we present a very rare case of a difficult interpretation of the diagnosis of an arrhythmia in an HF patient with a CRT device with a defibrillator (CRT-D).

## Case report

**Figure 1:**
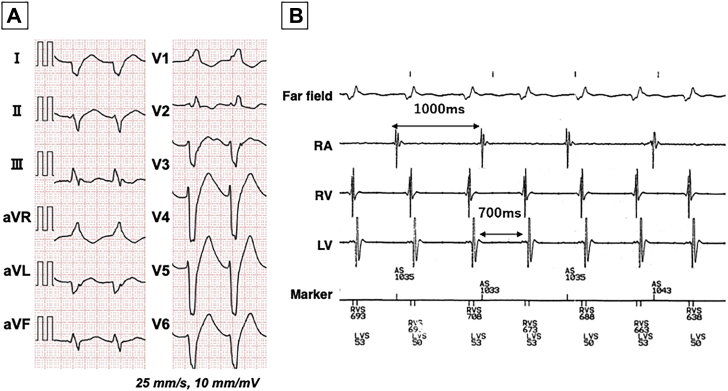
**A:** A 12-lead electrocardiogram during the wide QRS rhythm at 90 beats/min exhibited a QRS width of 200 ms, right bundle branch block pattern, and marked left QRS axis deviation without any obvious P waves. **B:** The telemetry tracings from the device during the wide QRS rhythm exhibited a V-V interval of 700 ms that was shorter than the A-A interval from the right atrium (RA). LV = left ventricle; RV = right ventricle.

A 66-year-old man with familial dilated cardiomyopathy and a CRT-D device (Resonate X4; Boston Scientific, Marlborough, MA) visited our outpatient clinic owing to transient faintness and palpitations. He had been diagnosed with juvenile sick sinus syndrome and was implanted with a permanent pacemaker at the age of 25. Furthermore, his son had been diagnosed with juvenile dilated cardiomyopathy and died at the age of 15. The pulse rate was 90 beats per minute and systolic blood pressure 102 mm Hg. The 12-lead electrocardiogram (ECG) revealed a regular wide QRS rhythm with a QRS duration of 200 ms and right bundle branch block configuration with a marked left axis deviation in the absence of obvious P waves (Figure 1A). The device interrogation demonstrated ventricular sensing without CRT pacing at a V-V interval of 700 ms, which was faster than the right atrial (RA) regular rhythm (Figure 1B). The ventricular rhythm was not diagnosed as a tachycardia by the CRT-D detection algorithm because the detection zone for ventricular tachyarrhythmias for defibrillation or antitachycardia pacing had been programmed to over 150 beats per minute. From those observations, our initial speculation was that it was an accelerated idioventricular rhythm (AIVR) with VA dissociation. Then, we decided to attempt a catheter ablation procedure because the AIVR inhibited effective CRT pacing.

**Figure 2:**
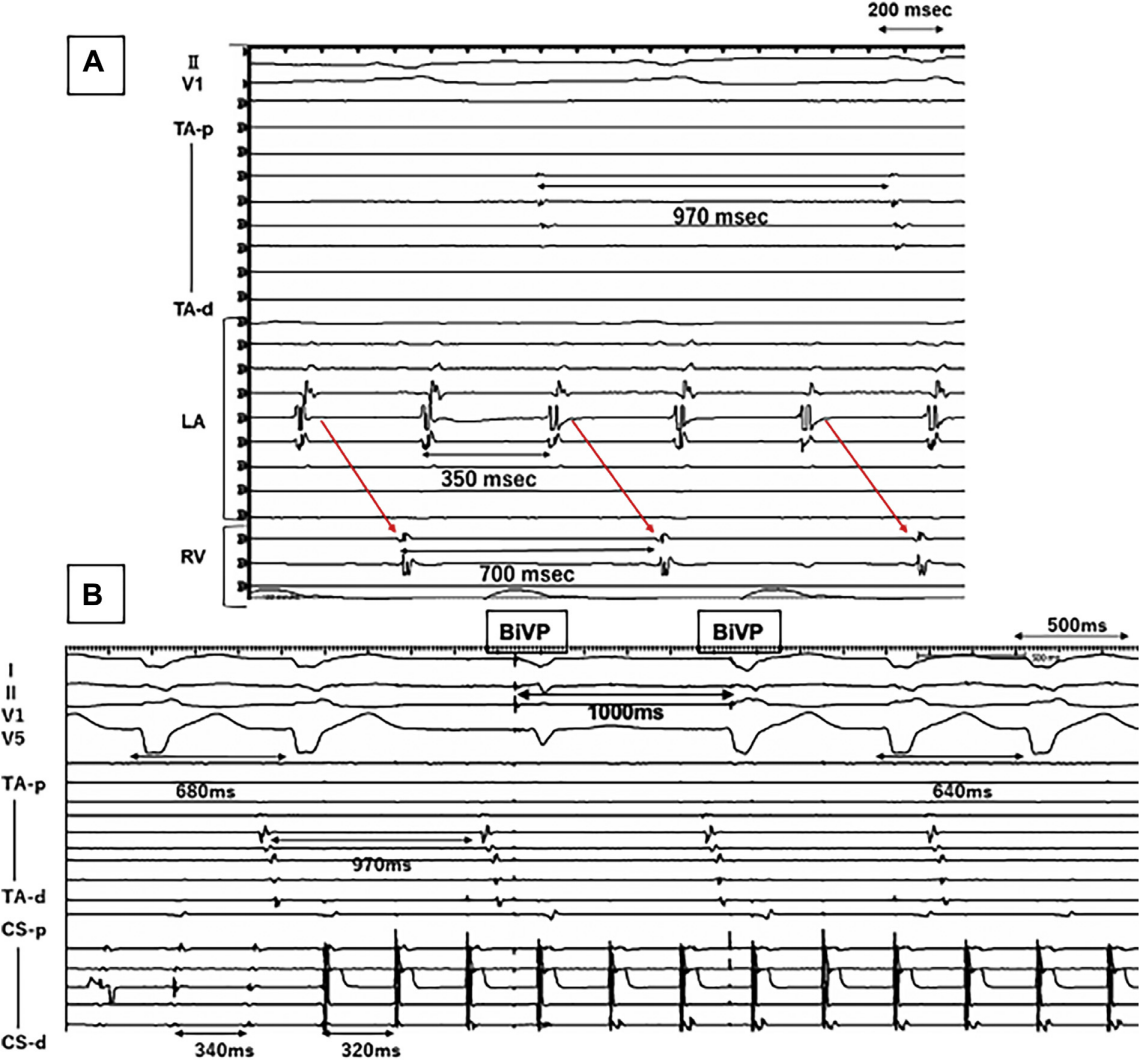
**A:** The intracardiac electrogram tracings from the right atrium (RA), left atrium (LA), and right ventricle (RV) during the wide QRS rhythm revealed the fastest rhythm was in the LA (A-A: 350 ms) and a 2-fold V-V interval (700 ms) due to 2:1 atrioventricular conduction. The slowest rhythm was identified in the RA, which was electrically dissociated from the LA. **B:** Overdrive pacing at 320 ms from the distal coronary vein (CV) induced a transient AV prolongation with biventricular pacing (BiVP) and then shortened the V-V interval to 640 ms. The RA rhythm was not affected by the CV pacing. CS-d = coronary sinus-distal; CS-p = coronary sinus-proximal; TA-d = tricuspid annulus-distal; TA-p = tricuspid annulus-proximal.

**Figure 3:**
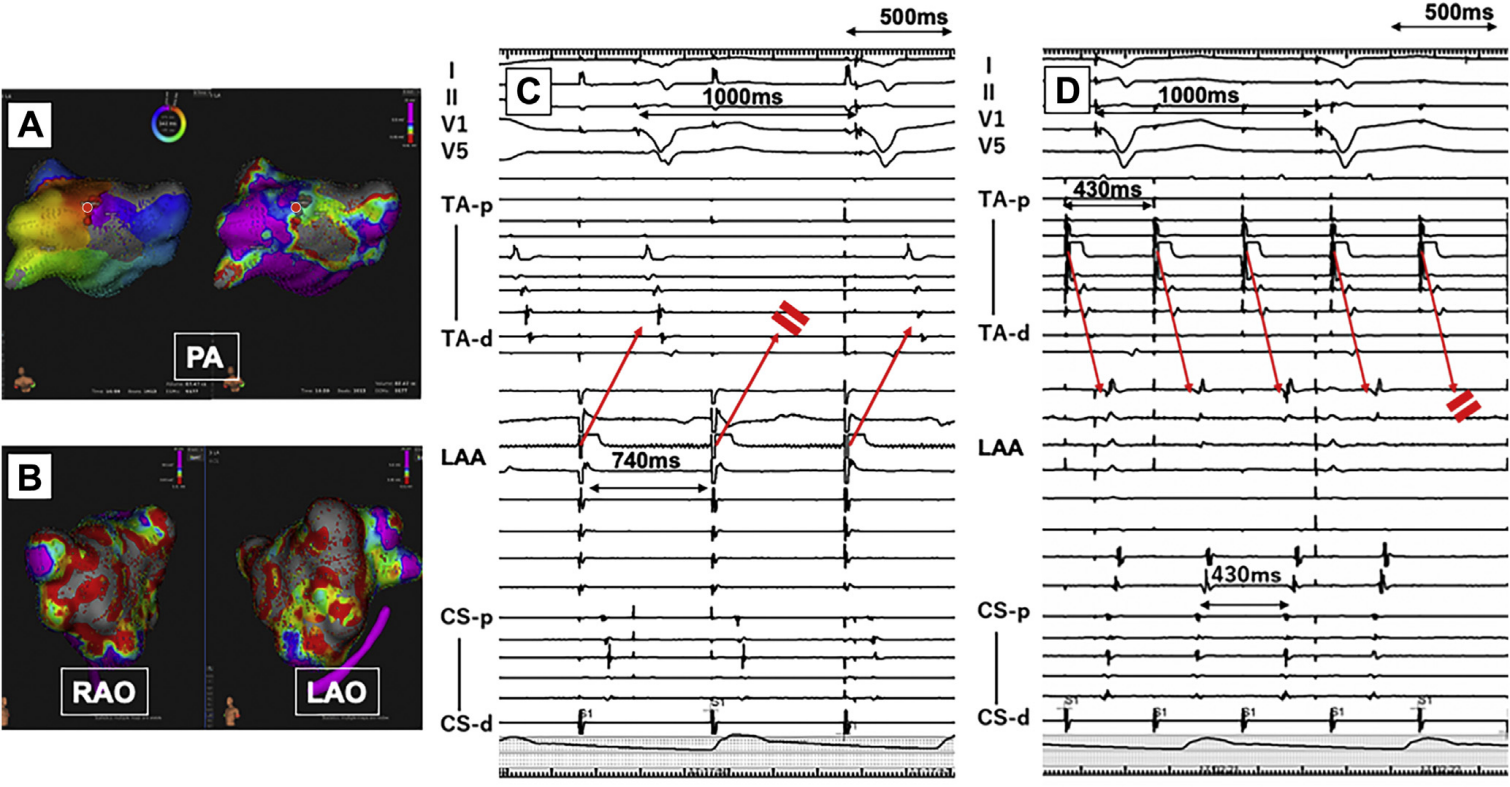
**A:** A 3-D electroanatomical activation map during the left atrial tachycardia (left panel) and voltage map (right panel) demonstrated a reentrant mechanism with a narrow channel in the low-voltage area in the posterior left atrium (LA). The successful ablation site is indicated by the red circles. **B:** Three-dimensional electroanatomical voltage maps demonstrating an extended low-voltage area on the left anterior wall. **C:** Left atrial appendage (LAA) pacing demonstrated LA-to–right atrium (RA) conduction block at a pacing cycle length of 740 ms. **D:** Tricuspid annulus pacing demonstrated 1:1 RA-to-LA conduction at pacing cycle lengths longer than 440 ms; however, Wenckebach-type RA-to-LA conduction block occurred at 430 ms. CS-d = coronary sinus-distal; CS-p = coronary sinus-proximal; LAO = left anterior oblique view; PA = posteroanterior view; RAO = right anterior oblique view; TA-d = tricuspid annulus-distal; TA-p = tricuspid annulus-proximal.

At the beginning of the procedure, a 10-polar catheter and a 20-polar catheter were placed into the coronary vein (CV) and on the RA free wall, respectively. A 4-polar catheter was also placed in the right ventricular apex. The intracardiac electrograms revealed different A-A intervals between the right atrium (970 ms) and CV (350 ms) with a V-V interval of 700 ms (Figure 2A). Overdrive pacing at 320 ms from the distal CV induced a transient AV prolongation with biventricular pacing and then shortened the V-V interval to 640 ms. The RA rhythm was not affected by the CV pacing (Figure 2B). Our final rhythm diagnoses were a left atrial tachycardia (AT) with simultaneous sinus rhythm observed in the RA. Interatrial dissociation (LA-to-RA conduction block) with 2:1 LA-to-V conduction, right bundle branch block, and marked left axis deviation of the conducted QRS complex were observed. Three-dimensional electroanatomical mapping of the LA during the AT was performed using a Rhythmia® system (Boston Scientific, Marlborough, MN). Activation mapping revealed a reentrant tachycardia pattern with a head-meets-tail-style activation at the center of the LA roof (Figure 3A) and voltage mapping revealed low-voltage areas around the anterior wall and roof of the LA (Figure 3B). A radiofrequency ablation catheter was inserted into the LA by the trans–atrial septal approach, and the left AT was successfully eliminated by a single radiofrequency application at a left high posterior LA site where diastolic fragmented potentials were recorded. The CRT pacing was restored immediately after the termination of the left AT. Then, we examined the interatrial conduction by a programmed stimulation method from the LA appendage and tricuspid annulus. The LA-to-RA conduction blocked at a pacing cycle length of 740 ms (Figure 3C) and 1:1 RA-to-LA conduction was observed up to a pacing cycle length longer than 440 ms; however, Wenckebach-type block occurred at 430 ms. The activation sequence of the coronary sinus was “distal to proximal,” which suggested that the RA-to-LA conduction was dominant through the Bachmann bundle region rather than the mid–atrial septum or coronary sinus region (Figure 3D). A single extrastimulation with a basic pacing cycle of 700 ms from the high RA resulted in RA-to-LA conduction block at 350 ms, and stimulation from the LA appendage exhibited LA-to-RA block at 440 ms. These findings suggested rate-dependent, bidirectional interatrial conduction block with a greater impaired conduction in the LA-to-RA direction than RA-to-LA direction, resulting in interatrial dissociation only during the LA tachycardia. After the successful ablation of the left AT, regular CRT pacing resumed immediately and was maintained during 20 months of follow-up.

## Discussion

We had made a false diagnosis of AIVR from the CRT-D telemetry data (a wide QRS rhythm with a V-V interval shorter than the right A-A interval) at the beginning because our case represented extremely rare electrophysiological findings: (1) interatrial dissociation; (2) a sustained left AT, which was not detected by the telemetry ECG tracing or 12-lead ECG; and (3) 2:1 LA-to-V conduction without RA-to-V conduction.

There are numerous algorithms and criteria for discriminating wide QRS rhythms based on the 12-lead ECG findings.^5^ A wide QRS complex (≥140 ms), marked QRS axis deviation, RS pattern in V_6_ lead,^6^ or positive concordance in aVR lead^7^ may be interpreted as a ventricular rhythm. In our case, the 12-lead ECG during the arrhythmia demonstrated a QRS width of 200 ms, QRS axis of -170 degrees, and monophasic R pattern in aVR (Figure 1). Moreover, VA dissociation during the arrhythmia was indicated from the CRT-D telemetry findings.

However, once the information of the LA was obtained from the coronary sinus catheter, we could reach a correct diagnosis; that is, the wide QRS rhythm we had believed to be AIVR was actually a left AT with interatrial conduction block from the LA to RA with 2:1 LA-to-V conduction. According to the previous reports, the interatrial connections are made up of the Bachmann bundle, the coronary sinus musculature, and transseptal connections^8^; moreover, the disturbance of these connections may cause atrial arrhythmias.^9^ Sakamoto and colleagues^10^ reported a case with 2 different morphologies of an AT owing to a single left AT mechanism with an interatrial conduction disturbance. Markowitz and colleagues^11^ reported 2 patients associated with interatrial and AV conduction block after a successful right AT ablation in a case series of an unintentional regional isolation caused by an RA ablation. In our case, an anisotropic LA-to-RA and RA-to-LA conduction disturbance and also an anisotropic LA-to-V and RA-to-V conduction were observed. These findings may be explained by the complex fibrotic lesions in the bilateral atria and around the perinodal tissue.^12^^,^^13^

Atrial remodeling is common in patients with advanced structural heart disease.^14^ The current algorithms for differentiating ventricular arrhythmias using the 12-lead ECG or intracardiac tracing of cardiac implantable electronic devices may not warrant a correct diagnosis in a specific population.

## Conclusion

We experienced a case with a unique mechanism of a wide QRS arrhythmia: a left AT with an interatrial conduction disturbance and 2:1 LA-to-V conduction in a patient with a CRT-D. We should note that the current algorithms for a differential diagnosis of wide QRS arrhythmias cannot always provide a correct diagnosis, especially in patients with advanced myocardial degeneration. Abnormal electrical activations in both the RA and left atrium should be taken into account to understand the true mechanism.

**Key Teaching Points**

• The discrimination of wide QRS arrhythmias is sometimes hard to achieve based on the existing criterion of the 12-lead electrocardiogram or intracardiac electrogram findings, especially in heart failure patients.
• Interatrial conduction disturbances possibly make the left atrial electrical activity indeterminable in patients with cardiac resynchronization therapy.
• The right atrium-to-ventricle conduction could also dissociate from the left atrium-to-ventricle conduction, possibly based on advanced myocardial degeneration.
